# Entropy, Uncertainty, and the Depth of Implicit Knowledge on Musical Creativity: Computational Study of Improvisation in Melody and Rhythm

**DOI:** 10.3389/fncom.2018.00097

**Published:** 2018-12-19

**Authors:** Tatsuya Daikoku

**Affiliations:** Department of Neuropsychology, Max Planck Institute for Human Cognitive and Brain Sciences, Leipzig, Germany

**Keywords:** creativity, Markov model, N-gram, improvisation, statistical learning, machine learning, uncertainty, entropy

## Abstract

Recent neurophysiological and computational studies have proposed the hypothesis that our brain automatically codes the *n*th-order transitional probabilities (TPs) embedded in sequential phenomena such as music and language (i.e., local statistics in *n*th-order level), grasps the entropy of the TP distribution (i.e., global statistics), and predicts the future state based on the internalized *n*th-order statistical model. This mechanism is called statistical learning (SL). SL is also believed to contribute to the creativity involved in musical improvisation. The present study examines the interactions among local statistics, global statistics, and different levels of orders (mutual information) in musical improvisation interact. Interactions among local statistics, global statistics, and hierarchy were detected in higher-order SL models of pitches, but not lower-order SL models of pitches or SL models of rhythms. These results suggest that the information-theoretical phenomena of local and global statistics in each order may be reflected in improvisational music. The present study proposes novel methodology to evaluate musical creativity associated with SL based on information theory.

## Introduction

### Statistical Learning in the Brain: Local and Global Statistics

The notion of statistical learning (SL) (Saffran et al., 1996), which includes both informatics and neurophysiology (Harrison et al., 2006; Pearce and Wiggins, 2012), involves the hypothesis that our brain automatically codes the *n*th-order transitional probabilities (TPs) embedded in sequential phenomena such as music and language (i.e., local statistics in *n*th-order levels) (Daikoku et al., 2016, 2017b,c; Daikoku and Yumoto, 2017), grasps the entropy/uncertainty of the TP distribution (i.e., global statistics) (Hasson, 2017), predicts the future state based on the internalized *n*th-order statistical model (Daikoku et al., 2014; Yumoto and Daikoku, 2016), and continually updates the model to adapt to the variable external environment (Daikoku et al., 2012, 2017d). The concept of brain *n*th-order SL is underpinned by information theory (Shannon, 1951) involving *n*-gram or Markov models. TP (local statistics) and entropy (global statistics) are used to estimate the statistical structure of environmental information. The *n*th-order Markov model is a mathematical system based on the conditional probability of sequence in which the probability of the forthcoming state is statistically defined by the most recent *n* state (i.e., *n*th-order TP). A recent neurophysiological study suggested that sequences with higher entropy are learned based on higher-order TP whereas those with lower entropy are learned based on lower-order TP (Daikoku et al., 2017a). Another study suggested that certain regions or networks perform specific computations of global statistics (i.e., entropy) that are independent of local statistics (i.e., TP) (Hasson, 2017). Few studies, however, have investigated how perceptive systems of local and global statistics interact. It is important to examine the entire process of brain SL in both computational and neurophysiological areas (Daikoku, 2018b).

### Statistical Learning and Information Theory

#### Local Statistics: Nth-Order Transitional Probability

**Figure 1:**
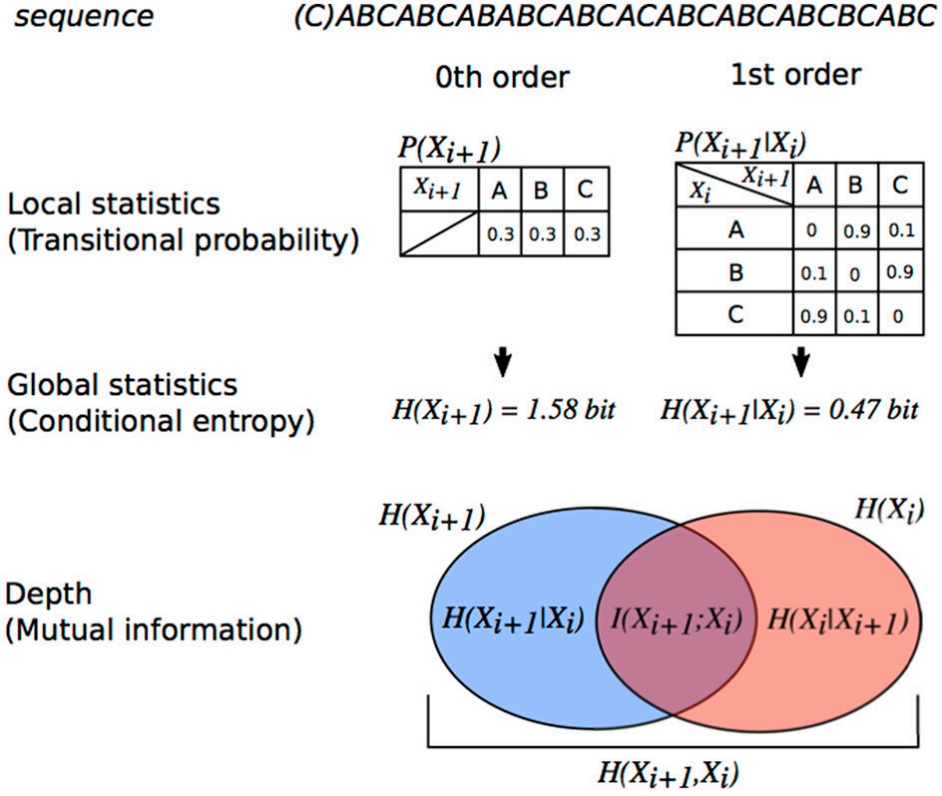
Relationship between order of transitional probabilities, entropy, conditional entropy, and MI illustrated using a Venn diagram. The degree of dependence on X_i_ for X_i+1_ is measured by MI (MI (*I(X;Y)*) = entropy (*H(X*_*i*+1_*)*] – conditional entropy [*H(X*_*i*+1_*|X*_*i*_*)*)). The MI of sequences in this figure is more than 0. Thus, each event X_i+1_ in the sequence is dependent on a preceding event *X*_*i*_.

Research suggests that there are two types of coding systems involved in brain SL (see Figure 1): *n*th-order TPs (local statistics at various order levels) (Daikoku et al., 2017a; Daikoku, 2018a) and uncertainty/entropy (global statistics) (Hasson, 2017). The TP is the conditional probability of an event B, given that the most recent event A has occurred—this is written as P(B|A). The nth-order TP distributions sampled from sequential information such as music and language can be expressed by *n*th-order Markov models (Markov, 1971). The *n*th-order Markov model is based on the conditional probability of an event *e*_*n*+1_, given the preceding *n* events based on Bayes' theorem [*P(e*_*n*+1_*|e*_*n*_*)*]. From a psychological viewpoint, the formula can be interpreted as positing that the brain predicts a subsequent event *e*_*n*+1_ based on the preceding events *e*_*n*_ in a sequence. In other words, learners expect the event with the highest TP based on the latest n states, and are likely to be surprised by an event with lower TP. Furthermore, TPs are often translated as information contents [ICs, *-log*_2_*1/P(e*_*n*+1_*|e*_*n*_*)*], which can be regarded as degrees of surprising and predictable (Pearce and Wiggins, 2006). A lower IC (i.e., higher TPs) means higher predictability and smaller surprise whereas a higher IC (i.e., lower TPs) means lower predictability and larger surprise. In the end, a tone with lower IC may be one that a composer is more likely to predict and choose as the next tone compared to tones with higher IC. IC can be used in computational studies of music to discuss the psychological phenomena involved in prediction and SL.

#### Global Statistics: Entropy and Uncertainty

Entropy (i.e., global statistics, Figure 1) is also used to understand the general predictability of a sequence (Manzara et al., 1992; Reis, 1999; Cox, 2010). It is calculated from probability distribution, interpreted as uncertainty (Friston, 2010), and used to evaluate the neurophysiological effects of global SL (Harrison et al., 2006) as well as decision making (Summerfield and de Lange, 2014), anxiety (Hirsh et al., 2012), and curiosity (Loewenstein, 1994). A previous study reported that the neural systems of global SL were partially independent of those of local SL (Hasson, 2017). Furthermore, reorganization of learned local statistics requires more time than the acquisition of new local statistics, even if the new and previously acquired information sets have equivalent entropy levels (Daikoku et al., 2017d). Some articles, however, suggest that the global statistics of sequence modulate local SL (Daikoku et al., 2017a). Furthermore, uncertainty of auditory and visual statistics is coded by modality-general, as well as modality-specific, neural systems (Strange et al., 2005; Nastase et al., 2014). This suggests that the neural basis that codes global statistics, as well as local statistics, is a domain-general system. Although domain-general and domain-specific learning system in the brain are under debate (Hauser et al., 2002; Jackendoff and Lerdahl, 2006), there seems to be neural and psychological interactions in perceptions between local and global statistics.

#### Depth: Mutual Information

Mutual information (MI) and pointwise MI (PMI) are measures of the mutual dependence between two variables. PMI refers to each event in sequence (local dependence), and MI refers to the average of all events in the sequence (global dependence). In the framework of SL based on TPs [*P(e*_*n*+1_*|e*_*n*_*)*], MI explains how an event *e*_*n*+1_ is dependent on the preceding event *e*_*n*_. Thus, MI is key to understanding the order of SL. For example, a typical oddball sequence consisting of a frequent stimulus with high probability of appearance and a deviant stimulus with low probability of appearance has weak dependence between two adjacent events (*e*_*n*_*, e*_*n*+1_) and shows low MI, because event *e*_*n*+1_ appears independently of the preceding events *e*_*n*_. In contrast, an SL sequence based on TPs, but not probabilities of appearance, has strong dependence on the two adjacent events and shows larger MI. For example, a typical SL paradigm that consists of the concatenation of pseudo-words with three stimuli has large MI until second-order Markov or tri-gram models [i.e., *P(C|AB)*] whereas it has low MI from third-order Markov or four-gram models [i.e., *P(D|ABC)*]. Thus, MI is sometimes used to evaluate levels of SL in both neurophysiological (Harrison et al., 2006) and computational studies (Pearce et al., 2010). In sum, the three types of information-theoretical evaluations of SL models (i.e., IC, entropy, and MI) can be explained in terms of psychological aspects. (1) IC reflects local statistics. A tone with lower IC (i.e., higher TPs) may be one that a composer is more likely to predict and choose as the next tone compared to tones with higher IC. (2) Entropy reflects global statistics and is interpreted as the uncertainty of whole sequences. (3) MI reflects the levels of orders in statistics and is interpreted as the dependence of preceding sequential events in SL. Using them, the present study investigated how local statistics, global statistics, and the levels of the orders in musical improvisation interact.

### Musical Improvisation

Implicit statistical knowledge is considered to contribute to the creativity involved in musical composition and musical improvisation (Pearce and Wiggins, 2012; Norgaard, 2014; Wiggins, 2018). Additionally, it is widely accepted that implicit knowledge causes a sense of intuition, spontaneous behavior, skill acquisition based on procedural learning, and creativity, and is also closely tied to musical expression, such as composition, playing, and intuitive creativity. Particularly, in musical improvisation, musicians are forced to express intuitive creativity and immediately play their own music based on long-term training associated with procedural and implicit learning (Clark and Squire, 1998; Ullman, 2001; Paradis, 2004; De Jong, 2005; Ellis, 2009; Müller et al., 2016). Thus, compared to other types of musical composition in which a composer deliberates and refines a composition scheme for a long time based on musical theory, the performance of musical improvisation is intimately bound to implicit knowledge because of the necessity of intuitive decision making (Berry and Dienes, 1993; Reber, 1993; Perkovic and Orquin, 2017) and auditory-motor planning based on procedural knowledge (Pearce et al., 2010; Norgaard, 2014). This suggests that the stochastic distribution calculated from musical improvisation may represent the musicians' implicit knowledge and creativity in music that has been developed via implicit learning. Few studies have investigated the relationship between musical improvisation and implicit statistical knowledge. The present study, using real-world improvisational music, first proposed a computational model of musical creativity in improvisation based on TP distribution, and examined how local statistics, global statistics, and hierarchy in music interact.

## Methods

### Extraction of Spectral and Temporal Information

#### General Methodologies

The three musicians of William John Evans (Autumn Leaves from Portrait in Jazz, 1959; Israel from Explorations, February 1961; I Love You Porgy from Waltz for Debby, June 1961; Stella by Starlight from Conversations with Myself, 1963; Who Can I Turn To? from Bill Evans at Town Hall, 1966; Someday My Prince Will Come from the Montreux Jazz Festival, 1968; A Time for Love from Alone, 1969), Herbert Jeffrey Hancock (Cantaloupe Island from Empyrean Isles, 1964; Maiden Voyage from Flood, 1975; Someday My Prince Will Come from The Piano, 1978; Dolphin Dance from Herbie Hancock Trio '81, 1981; Thieves in the Temple from The New Standard, 1996; Cottontail from Gershwin's World, 1998; The Sorcerer from Directions in Music, 2001), and McCoy Tyner (Man from Tanganyika from Tender Moments, 1967; Folks from Echoes of a Friend, 1972; You Stepped Out of a Dream from Fly with the Wind, 1976; For Tomorrow from Inner Voice; 1977; The Habana Sun from The Legend of the Hour, 1981; Autumn Leaves from Revelations, 1988; Just in Time from Dimensions, 1984) were used in the present study. The highest pitches with length were extracted based on the following definitions: the highest pitches that can be played at a given point in time, pitches with slurs that can be counted as one, and grace notes were excluded. In addition, the rests that were related to highest-pitch sequences were also extracted. This spectral and temporal information were divided into four types of sequences: [1] a pitch sequence without length and rest information (i.e., pitch sequence without temporal information); [2] a temporal sequence without pitch information (i.e., temporal sequence without pitches); [3] a pitch sequence with length and rest information (i.e., pitch sequence with temporal information); and [4] a temporal sequence with pitch information (i.e., temporal sequence with pitches).

#### Pitch Sequence Without Temporal Information

**Figure 2:**
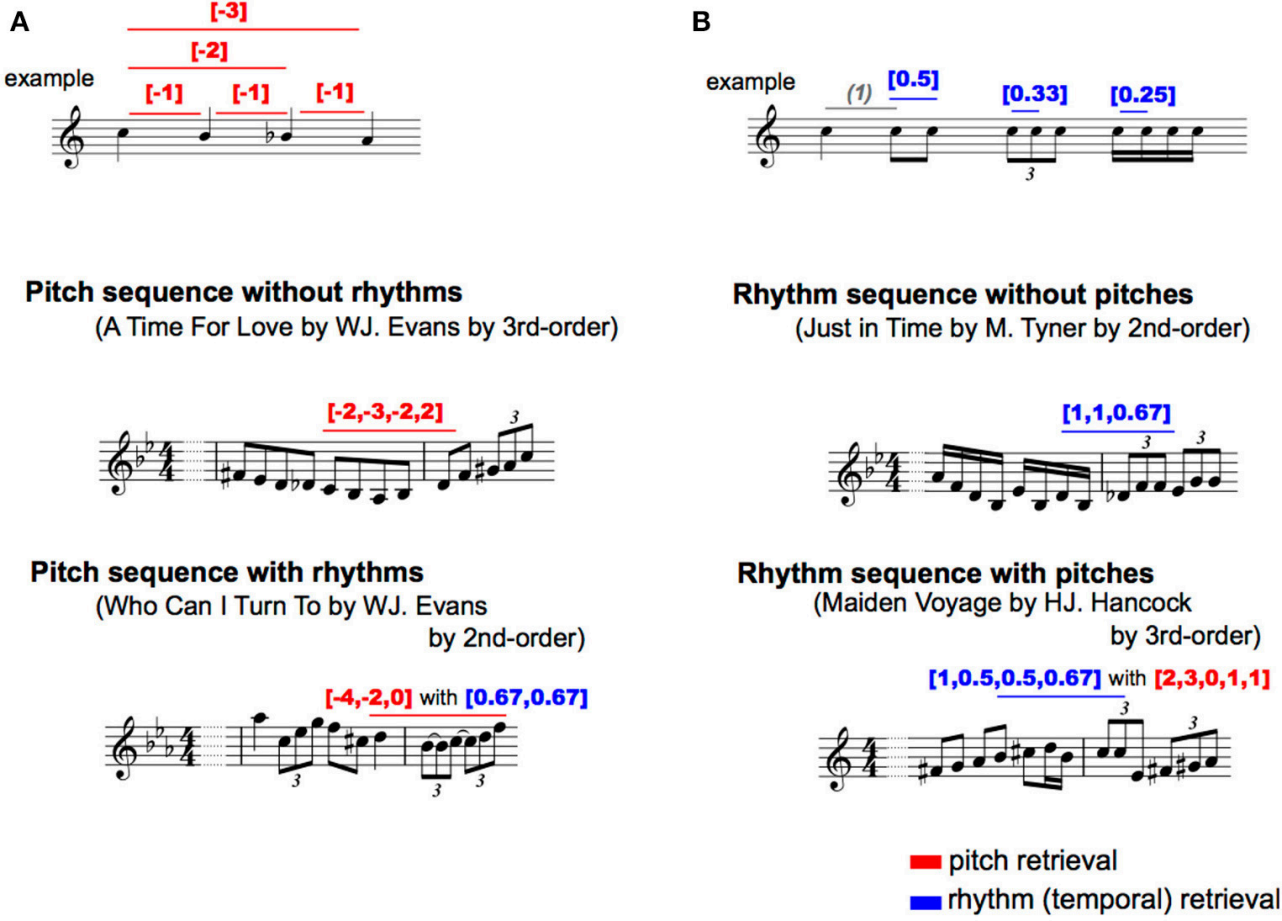
Representative phrases of each type of transition pattern. Red: pitch transition, Blue: rhythm (temporal) transition. **(A)** Pitch**; (B)** Rhythm.

For each type of pitch sequence, all of the intervals were numbered so that an increase or decrease in a semitone was 1 and −1 based on the first pitch, respectively. Representative examples were shown in Figure 2. This revealed the relative pitch-interval patterns but not the absolute pitch patterns. This procedure was used to eliminate the effects of the change in key on transitional patterns. Interpretation of the key change depends on the musician, and it is difficult to define in an objective manner. Thus, the results in the present study may represent a variation in the statistics associated with relative pitch rather than absolute pitch.

#### Temporal Sequence Without Pitches

The onset times of each note were used for analyses. Although, note onsets ignore the length of notes and rests, this methodology can capture the most essential rhythmic features of the music (Povel, 1984; Norgaard, 2014). To extract a temporal interval between adjacent notes, all onset times were subtracted from the onset of the preceding note. Then, for each type of temporal sequence, the second to last temporal interval was divided by the first temporal interval. Representative examples are shown in Figure 2. This revealed relative rhythm patterns but not absolute rhythm patterns; it is independent of the tempo of each piece of music.

#### Pitch Sequence With Temporal Information

The two methodologies of pitch and temporal sequences were combined. For each type of sequence, all of the intervals were numbered so that an increase or decrease in a semitone was 1 and −1 based on the first pitch, respectively. Additionally, for each type of pitch sequence, all onset times were subtracted from the onset of the preceding note, and the second to last temporal intervals were divided by the first temporal interval. The representative examples were shown in Figure 2. On the other hand, a temporal interval of first-order model was calculated as a ratio to the crotchet (i.e., quarter note), because only a temporal interval is included for each sequence and the note length cannot be calculated as a relative temporal interval. Thus, the patterns of pitch sequence (*p*) with temporal information (*t*) were represented as [*p*] with [*t*].

#### Temporal Sequence With Pitches

The methodologies of sequence extraction were the same as those of the pitch sequence with rhythm (see Figure 2), whereas the TPs of the rhythm, but not pitch, sequences were calculated as a statistic based on multi-order Markov chains. The probability of a forthcoming temporal interval with pitch was statistically defined by the last temporal interval with pitch to six successive temporal interval with pitch (i.e., first- to six-order Markov chains). Thus, the relative pattern of temporal sequence (*r*) with pitches (*p*) were represented as [*t*] with [*p*].

### Modeling and Analysis

The TPs of the sequential patterns were calculated based on 0th−5th-order Markov chains. The *n*th-order Markov chain is the conditional probability of an event e_n+1_, given the preceding *n* events based on Bayes' theorem:

(1) $$\begin{array}{r} P\left(e_{n+1}|e_{n}\right)\;=\;\frac{P\left(e_{n+1}\cap e_{n}\right)}{P\left(e_{n}\right)} \end{array}$$

The ICs (*I[e*_*n*+1_*|e*_*n*_*]*) and conditional entropy [*H(B|A)*] in the *n*th-order TP distribution (hereafter, Markov entropy) were calculated using TPs in the framework of information theory.

(2) $$\begin{array}{rcl} I\left(e_{n+1}|e_{n}\right) & = & \log_{2}\;\frac{1}{P\left(e_{n+1}|e_{n}\right)}\;\left(bit\right) \end{array}$$

(3) $$\begin{array}{rcl} H\left(B|A\right) & = & {-\sum }_{i}\;\underset{j}{\sum }P\left(ai\right)P\left(bj|ai\right)\;\log_{2}\;P\left(bj|ai\right)\;\left(bit\right) \end{array}$$

where *P(*bj|ai*)* is a conditional probability of sequence “*ai bj.”* Then, MI [*I(X;Y)*] were calculated in 1st-, 2nd-, and 3rd-order Markov models. MI is an information theoretic measure of dependency between two variables (Cover and Thomas, 1991). The MI of two discrete variables X and Y can be defined as

(4) $$\begin{array}{r} I\left(X;Y\right)\;=\;\sum_{y\in Y}\sum_{x\in X}p\left(x,y\right)\log\left(\frac{p\left(x,y\right)}{p\left(x\right)p\left(y\right)}\right)\;\left(bit\right) \end{array}$$

where *p*(x,y) is the joint probability function of X and Y, and *p*(x) and *p*(y) are the marginal probability distribution functions of X and Y, respectively. From entropy values, the MI can also be expressed as

(5) $$\begin{array}{lll} I\left(X;Y\right) & = & \;\sum_{x,y}p\left(x,y\right)\log\left(\frac{p\left(x,y\right)}{p\left(x\right)p\left(y\right)}\right)\\ & = & \;\sum_{x,y}p\left(x,y\right)\log\left(\frac{p\left(x,y\right)}{p\left(x\right)}\right)-\sum_{x,y}p\left(x,y\right)\log p\left(y\right)\\ & = & \;\sum_{x,y}p\left(x\right)p\left(y|x\right)\log p\left(y|x\right)-\sum_{x,y}\log p\left(y\right)p\left(x,y\right)\\ & = & \;\sum_{x}p\left(x\right)\left(\sum_{y}p\left(y|x\right)\log p\left(y|x\right)\right)\\ & -\sum_{y}\log p\left(y\right)\left(\sum_{x}p\left(x,y\right)\right)\\ & = & \;-\sum_{x}p\left(x\right)H\left(Y|X=x\right)-\sum_{y}p\left(y\right)\log p\left(y\right)\\ & = & \;-H\left(Y|X\right)+\;H\left(Y\right)\\ & = & \;H\left(Y\right)-H\left(Y|X\right)\;\left(bit\right) \end{array}$$

where H(X) and H(Y) are the marginal entropies, H(X|Y) and H(Y|X) are the conditional entropies, and H(X,Y) is the joint entropy of X and Y (Figure 1). Based on psychological and information-theoretical concepts, the Equation (5) can be regarded that the amount of entropy (uncertainty) remaining about Y after X is known. That is, the MI is corresponding to reduction in entropy (uncertainty). Then, the transitional patterns with 1st−20th highest TPs in all musicians, which show higher predictabilities in each musician, were used as local statistics of familiar phrases. The applied familiar phrases and the TPs were shown in Supplementary material. The TPs of familiar phrases were averaged. Repeated-measure analysis of variances (ANOVAs) with factors of order and type of sequence were conducted in each IC, entropy, and MI. Furthermore, the global statistics and MI in each order were compared with local statistics of familiar phrases by Pearson's correlation analysis. Statistical significance levels were set at *p* = 0.05 for all analyses.

## Results

### Local vs. Global Statistics

**Figure 3:**
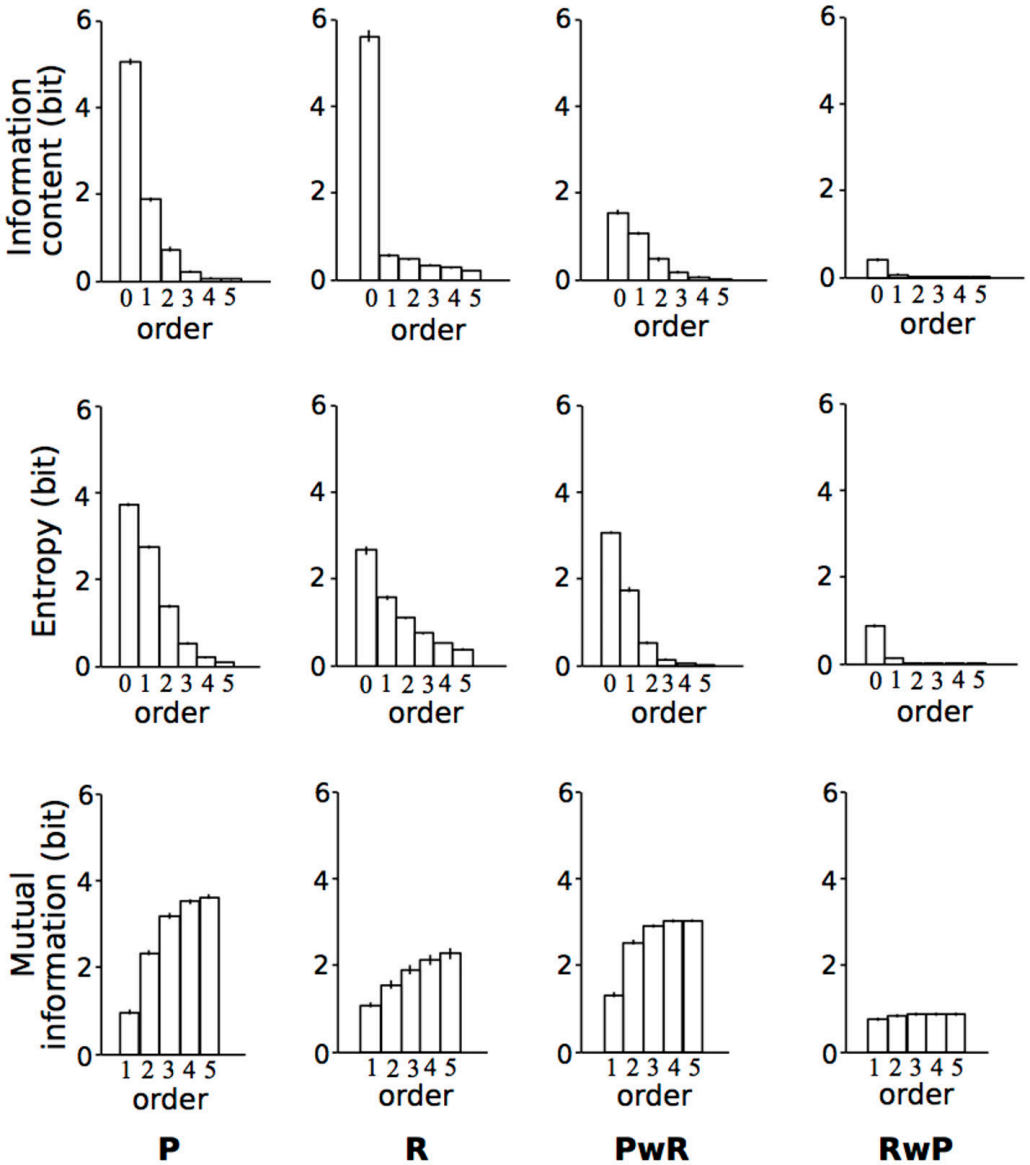
The means of information content (IC), Conditional entropy, and mutual information (MI). Error bars represent standard errors of the means. P, pitch sequence; R, rhythm sequence; PwR, pitch sequence with rhythms; RwP, rhythm sequence with pitches.

The means of IC, conditional entropy, and mutual information were shown in Figure 3. The means of IC, conditional entropy, and mutual information were shown in Figure 3. The main sequence effect were significant [IC: *F*_(2.39, 47.89)_ = 1010.07, *p* < 0.001, partial η^2^ = 0.98; Entropy: *F*_(1.20, 23.92)_ = 828.82, *p* < 0.001, partial η^2^ = 0.98; MI: *F*_(2.00, 39.91)_ = 225.54, *p* < 0.001, partial η^2^ = 0.92] (Table 1). The main order effect were significant [IC: *F*_(2.05, 40.93)_ = 2909.59, *p* < 0.001, partial η^2^ = .99; Entropy: *F*_(1.55, 31.03)_ = 2166.02, *p* < 0.001, partial η^2^ = 0.99; MI: *F*_(1.68, 33.59)_ = 2468.35, *p* < 0.001, partial η^2^ = 0.99] (Table 1). The order-sequence interactions were significant [IC: *F*_(3.39, 67.76)_ = 592.24, *p* < 0.001, partial η^2^ = 0.97; Entropy: *F*_(2.25, 44.94)_ = 282.95, *p* < 0.001, partial η^2^ = 0.93; MI: *F*_(1.82, 36.45)_ = 351.48, *p* < 0.001, partial η^2^ = 0.95)] (Table 1).

**Table 1:** ANOVA results.

**A. MAIN EFFECT**								
			**IC**		**Entrophy**		**MI**	
			**Diff**	***p*-value**	**Diff**	***p*-value**	**Diff**	***p*-value**
Sequence	P	R	0.09	0.043	0.28	<0.001	0.94	<0.001
		PwR	0.78	<0.001	0.51	<0.001	0.16	0.021
		RwP	1.25	<0.001	1.27	<0.001	1.89	<0.001
	R	PwR	0.69	<0.001	0.23	<0.001	−0.78	<0.001
		RwP	1.16	<0.001	0.99	<0.001	0.95	<0.001
	PwR	RwP	0.47	<0.001	0.76	<0.001	1.72	<0.001
Order	0th	1st	2.26	<0.001	1.04	<0.001	
		2nd	2.73	<0.001	1.83	<0.001	
		3rd	2.97	<0.001	2.23	<0.001	
		4th	3.05	<0.001	2.39	<0.001	
		5th	3.09	<0.001	2.46	<0.001	
	1st	2nd	0.47	<0.001	0.79	<0.001	−0.79	<0.001
		3rd	0.71	<0.001	1.19	<0.001	−1.19	<0.001
		4th	0.79	<0.001	1.35	<0.001	−1.35	<0.001
		5th	0.83	<0.001	1.42	<0.001	−1.42	<0.001
	2nd	3rd	0.24	<0.001	0.4	<0.001	−0.4	<0.001
		4th	0.33	<0.001	0.56	<0.001	−0.56	<0.001
		5th	0.36	<0.001	0.64	<0.001	−0.64	<0.001
	3rd	4th	0.09	<0.001	0.16	<0.001	−0.16	<0.001
		5th	0.12	<0.001	0.24	<0.001	−0.24	<0.001
	4th	5th	0.03	0.001	0.07	<0.001	−0.07	<0.001
**B. INTERACTION**								
			**IC**		**Entropy**		**MI**	
**Order**	**Sequence**		**Diff**	***p*****-value**	**Diff**	***p*****–value**	**Diff**	***p*****-value**
0th	P	R	−0.54	0.009	1.06	<0.001	
		PwR	3.51	<0.001	0.65	<0.001	
		RwP	4.66	<0.001	2.84	<0.001	
	R	PwR	4.05	<0.001	−0.42	0.016	
		RwP	5.24	<0.001	1.78	<0.001	
	PwR	RwP	1.15	<0.001	2.2	<0.001	
1st	P	R	1.33	<0.001	1.18	<0.001	−0.120	0.356
		PwR	0.83	<0.001	1	<0.001	−0.35	<0.001
		RwP	1.82	<0.001	2.63	<0.001	0.22	0.039
	R	PwR	−0.56	<0.001	−0.180	0.525	−0.23	0.002
		RwP	0.49	<0.001	1.44	<0.001	0.34	<0.001
	PwR	RwP	0.99	<0.001	1.63	<0.001	0.57	<0.001
2nd	P	R	0.27	<0.001	0.28	<0.001	0.79	<0.001
		PwR	0.26	<0.001	0.84	<0.001	−0.19	0.032
		RwP	0.71	<0.001	1.34	<0.001	1.5	<0.001
	R	PwR	−0.010	1.000	0.56	<0.001	−0.98	<0.001
		RwP	0.44	<0.001	1.06	<0.001	0.71	<0.001
	PwR	RwP	0.45	<0.001	0.51	<0.001	1.69	<0.001
3rd	P	R	−0.12	0.022	−0.22	<0.001	1.28	<0.001
		PwR	0.050	0.772	0.37	<0.001	0.27	0.002
		RwP	0.22	<0.001	0.52	<0.001	2.32	<0.001
	R	PwR	0.16	0.011	0.59	<0.001	−107	<0.001
		RwP	0.33	<0.001	0.74	<0.001	1.04	<0.001
	PwR	RwP	0.17	<0.001	0.15	<0.001	2.05	<0.001
4th	P	R	−0.21	<0.001	−0.33	<0.001	1.39	<0.001
		PwR	0.000	1.000	0.15	<0.001	0.5	<0.001
		RwP	0.06	0.004	0.2	<0.001	2.64	<0.001
	R	PwR	0.21	<0.001	0.47	<0.001	−0.89	<0.001
		RwP	0.28	<0.001	0.53	<0.001	1.25	<0.001
	PwR	RwP	0.06	0.011	0.06	<0.001	2.14	<0.001
5th	P	R	−0.17	<0.001	−0.3	<0.001	1.36	<0.001
		PwR	0.03	0.027	0.06	<0.001	0.59	<0.001
		RwP	0.05	0.009	0.09	<0.001	2.75	<0.001
	R	PwR	0.2	<0.001	0.36	<0.001	−0.78	<0.001
		RwP	0.22	<0.001	0.39	<0.001	1.39	<0.001
	PwR	RwP	0.020	0.360	0.03	<0.001	2.17	<0.001

*IC, information content; MI, mutual information; Diff, mean difference*.

*P, pitch sequence; R, rhythm sequence; PwR, pitch sequence with rhythms; RwP, rhythm sequence with pitches*.

### Local vs. Global Statistics

**Figure 4:**
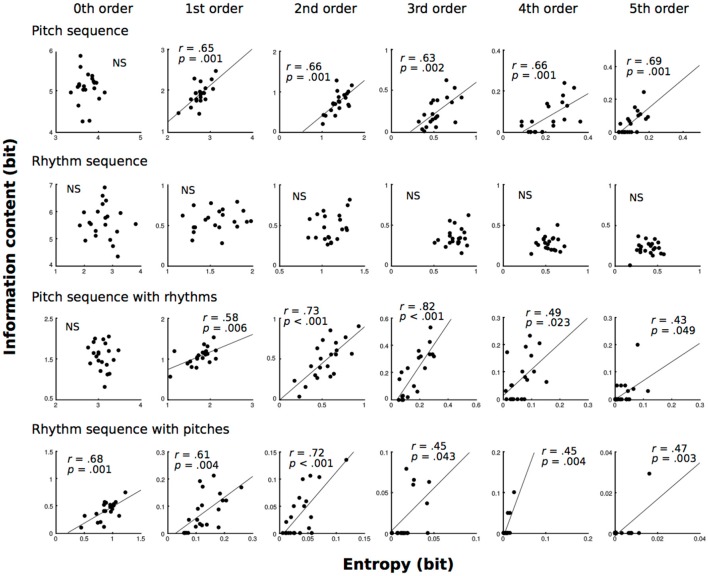
The correlation analysis between conditional entropy (global statistics) and ICs of familiar phrases (local statistics) based on zeroth- to fifth-order Markov models of pitch and temporal (rhythm) sequences.

All of the results in correlation analysis are shown in Figure 4. In pitch sequence without temporal information, 1st−5th-order models showed that the conditional entropies of the TP distributions were moderately (0.4 ≦ |r| < 0.7) related to the ICs of TPs of familiar phrases (1st: *r* = 0.65, *p* = 0.001; 2nd: *r* = 0.66, *p* = 0.001; 3rd: *r* = 0.63, *p* = 0.002; 4th: *r* = 0.66, *p* = 0.001; 5th: *r* = 0.69, *p* = 0.001). In pitch sequence with temporal information, 1st-, 4th, and 5th-order models showed that the conditional entropies of the TP distributions were moderately (0.4 ≦ |r| < 0.7) related to the ICs of TPs of familiar phrases (1st: *r* = 0.58, *p* = 0.006; 4th: *r* = 0.49, *p* = 0.023; 5th: *r* = 0.43, *p* = 0.049), and 2nd- and 3rd-order models showed that the conditional entropies of the TP distributions were strongly (0.7 ≦ |r| < 1.0) related to the ICs of TPs of familiar phrases (2nd: *r* = 0.73, *p* < 0.001; 3rd: *r* = 0.82, *p* < 0.001). In temporal sequence with pitches, 0th−5th-order models showed that the conditional entropies of the TP distributions were moderately (0.4 ≦ |r| < 0.7) related to the ICs of TPs of familiar phrases (0th: *r* = 0.68, *p* = 0.001; 1st: *r* = 0.61, *p* = 0.004; 2nd: *r* = 0.72, *p* < 0.001; 3rd: *r* = 0.45, *p* = 0.043; 4th: *r* = 0.45, *p* = 0.004; 5th: *r* = 0.47, *p* = 0.003).

### Local Statistics vs. Hierarchy

**Figure 5:**
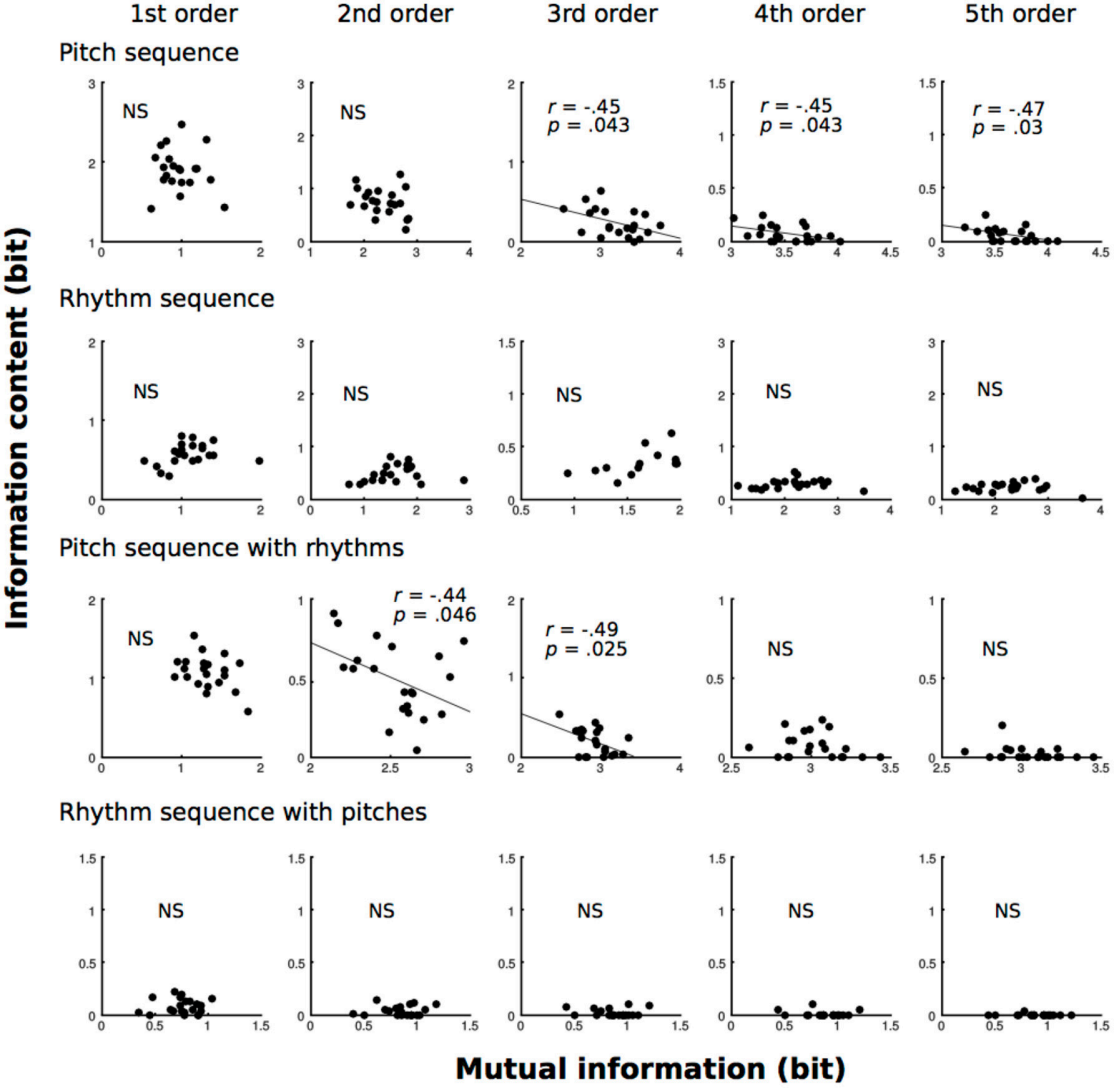
The correlation analysis between MI and ICs of familiar phrases (local statistics) based on zeroth- to fifth-order Markov models of pitch and temporal (rhythm) sequences.

All of the results are shown in Figure 5. In pitch sequence without temporal information, 3rd−5th-order models showed that the MI of the TP distributions were moderately (0.4 ≦ |r| < 0.7) related to the ICs of TPs of familiar phrases (3rd: *r* = 0.45, *p* = 0.043; 4th: *r* = 0.45, *p* = 0.043; 5th: *r* = 0.47, *p* = 0.03). In pitch sequence with temporal information, 2nd- and 3rd-order models showed that the MI of the TP distributions were moderately (0.4 ≦ |r| < 0.7) related to the ICs of TPs of familiar phrases (2nd: *r* = 0.44, *p* = 0.046; 3rd: *r* = 0.49, *p* = 0.025).

## Discussion

### Psychological Notions of Information Theory

The present study investigated how local statistics (TP and IC), global statistics (conditional entropy), and levels of orders (MI) in musical improvisation interact. The TP, IC, conditional entropy, and MI can be calculated based on Markov models, which are also applied to psychological and neurophysiological studies on SL (Harrison et al., 2006; Furl et al., 2011; Daikoku, 2018b). Based on psychological and neurophysiological studies on SL (Harrison et al., 2006; Pearce et al., 2010; de Zubicaray et al., 2013; Daikoku et al., 2015; Monroy et al., 2017), these three pieces of information can be translated to psychological indices: a tone with lower IC (i.e., higher TPs) may be one that a composer is more likely to predict and choose as the next tone compared to tones with higher IC whereas entropy and MI are interpreted as the global predictability of the sequences and the levels of order for the prediction, respectively. Previous studies also suggest that musical creativity in part depends on SL (Pearce, 2005; Pearce et al., 2010; Omigie et al., 2012, 2013; Pearce and Wiggins, 2012; Hansen and Pearce, 2014; Norgaard, 2014), and that musical training and experience is associated with the cognitive model of probabilistic structure in the music involved in SL (Pearce, 2005; Pearce and Wiggins, 2006; Pearce et al., 2010; Omigie et al., 2012, 2013; Pearce and Wiggins, 2012; Hansen and Pearce, 2014; Norgaard, 2014). The present study, using improvisational music by three musicians, examined how local and global statistics embedded in music interact, and discussed them from the interdisciplinary viewpoint of SL.

### Local vs. Global Statistics

In pitch sequence with and without temporal information, higher-order (1st−5th order) models detected positive correlations between global (conditional entropy) and local statistics (IC) in musical improvisation whereas no significance was detected in a lower-order (0th order) model. To understand the local statistics of familiar phrases, the present study used only the transitional patterns that showed the 1st−20th highest TPs for all musicians, which can be interpreted as higher predictabilities for each musician. Thus, the results suggest that, when the TPs of familiar phrases are decreased, the conditional entropy (uncertainty) of the entire TP distribution is increased. This finding is mathematically and psychologically reasonable. When improvisers are attempting to use various types of phrases, the variability of sequential patterns is increasing. In the end, the ICs (degree of surprise) of familiar phrases are positively correlated with the conditional entropy (uncertainty) of the entire sequential distribution. It is of note that this correlation could not be detected in a lower-order (0th order) model, and that no correlation was detected in a temporal sequence without pitches. This suggests that the interaction between local and global statistics may be stronger in the SL of spectral sequence compared to that of temporal sequence. Furthermore, these correlations may be detectable in higher-order models. This may suggest that higher-order SL can connect with grasping entropy. In sum, skills of musical improvisation and intuition may strongly depend on SL of pitch compared with that of rhythm. In addition, this phenomenon on intuition may occur in higher-, but not lower-order levels in SL. The higher-order SL model of pitches may be important to grasp the entire process of hierarchical SL in musical improvisation.

### Local Statistics vs. Hierarchy

In pitch sequences without temporal information, higher-order (3rd−5th order) models showed negative correlations between dependence of previous events (MI) and local statistics (IC), and no significance was detected in lower-order (0th−2nd order) models. This finding is also mathematically and psychologically reasonable. When players strongly depend on previous sequential information to improvise music, they tend to use familiar phrases because familiar phrases with higher TPs *P(X*_*i*+1_*|X*_*i*_*)* tend to have strong dependence on previous sequential information (*X*_*i*_). In the end, the ICs (degree of surprise) of familiar phrases are decreased when improvisers depend on previous sequential information that can be detected as larger MIs. Interestingly, this correlation could not be detected in a lower-order model (0th order), and no correlation was detected in the temporal sequence without pitches. As shown in the correlation between local and global statistics, the interaction between local statistics and levels of orders may be stronger in the SL of spectral sequence compared to that of temporal sequence. Furthermore, these correlations may be detectable in higher-order models. In contrast, fourth- and fifth-order models of pitch sequence with temporal information did not show correlations. Thus, rhythms may modulate the levels of orders in the SL of pitches in improvisational music (Daikoku, 2018c). This hypothesis may be supported in the models of temporal sequence with pitches. No correlation was detected in temporal sequence (Daikoku et al., 2018) with pitches. Future study is needed to investigate how rhythms affect improvisational music, and how the SL of rhythms interact with those of pitches. It is of note that the present study did not directly investigate the improviser's statistical knowledge of music, as only the statistics of music were analyzed. However, the transition probabilities shape only a small part of their respective styles. Future study should investigate the SL of music from many improvisers using interdisciplinary approaches of neurophysiology and informatics in parallel. The methodologies in this study are missing important information that constructs music such as beat, stresses, and ornamental note, which inspire the rhythm and intonation. Furthermore, the present study only analyzed three improvisers. To discuss universal phenomena in SL associated with improvisation, future study will be needed to examine a body of pieces of music.

## Conclusion

The present study investigated how local statistics (TP and IC), global statistics (entropy), and levels of orders (MI) in musical improvisation interact. Generally, the interactions among local statistics and global statistics were detected in higher-order SL models of pitches, but not lower-order SL models of spectral sequence or SL models of temporal sequence. The results of the present study suggested that information-theoretical phenomena of local and global statistics in each hierarchy can be reflected in improvisational music. These results support a novel methodology to evaluate musical creativity associated with SL based on information theory.

## Author Contributions

The author confirms being the sole contributor of this work and has approved it for publication.

### Conflict of Interest Statement

The author declares that the research was conducted in the absence of any commercial or financial relationships that could be construed as a potential conflict of interest.
